# Identification of angiotensin II-responsive circadian clock gene expression in adrenal zona glomerulosa cells and human adrenocortical H295R cells

**DOI:** 10.3389/fendo.2025.1525844

**Published:** 2025-03-26

**Authors:** Tomohiro Otani, Takahito Miyake, Takumi Ota, Daisuke Yarimizu, Yuuki Nakagawa, Iori Murai, Hitoshi Okamura, Emi Hasegawa, Masao Doi

**Affiliations:** ^1^ Department of Systems Biology, Graduate School of Pharmaceutical Sciences, Kyoto University, Kyoto, Japan; ^2^ Division of Physiology and Neurobiology, Graduate School of Medicine, Kyoto University, Kyoto, Japan

**Keywords:** chronobiology, circadian rhythm, zona glomerulosa, angiotensin II, candesartan, chronotherapy

## Abstract

The mammalian circadian timing system is organized in a hierarchy, with the master clock residing in the suprachiasmatic nucleus (SCN) of the hypothalamus and subsidiary peripheral clocks in peripheral tissues. Because of the diversity of peripheral tissues and cell-types in the body, the existence of autonomous clock and identification of its potential entrainment signals need to be empirically defined on a cell type-by-cell type basis. In this study, we characterized the basic circadian clock properties of the adrenal zona glomerulosa cells, or ZG cells. Using isolated adrenal explants from *Per2^Luc^* mice, dissociated ZG cells from *Per2-dluc* rats, and a related human adrenocortical cell line H295R, we showed that ZG cells possess genetically-encoded, self-sustained and cell-autonomous circadian clock. As to the potential entrainment signals, angiotensin II (Ang II) caused phase-dependent phase-shifts of adrenal ZG cells in cultured slices. Ang II treatment also drove initiation (or reset) of circadian clock gene expression in H295R cells with associated immediate up-regulation of *PER1* and *E4BP4* mRNA expression. We found that the type I Ang II receptor blocker CV11974, one of the most widely used clinical drugs for hypertensive diseases, caused attenuation of the phase resetting of H295R cells. Our *in vitro* data provide a basis to understand and argue for the adrenal gland ZG cells as a component of autonomous and entrainable peripheral clocks.

## 1 Introduction

Most aspects of physiology and behavior display circadian rhythms driven by an endogenous clock (1, 2). In mammals, a master pacemaker in the suprachiasmatic nucleus (SCN) regulates downstream oscillators in peripheral tissues (3–5). In all clock cell types, regardless of central vs. peripheral, the basic clock system is composed of three elementary elements: an oscillator that oscillates even under constant conditions; an input that enables the oscillator to synchronize with environmental cycles or internal time cues; and an output that transmits the oscillator’s signals into rhythmic gene expression and physiology, as referred to as the Eskinogram model (6). Within the oscillator, opposing effects of transcriptional activators and repressors constitute interlocked feedback loops (7–9). Briefly, the transcriptional activators CLOCK and BMAL1 drive transcription of *Period* (*Per1* and *Per2*) and *Cryptochrome* (*Cry1* and *Cry2*) via binding to E-box elements present in their promoters. Once the repressor proteins PER and CRY reach a critical concentration, they form complexes and repress their own expression by inhibiting the CLOCK/BMAL1 complexes. In addition to E-box elements, the promoter regions of *Per* genes contain D-boxes, through which the PAR bZip family of transcriptional activators (DBP, TEF and HLF) and the related repressor E4BP4 exert their opposing effects on the expression of target genes (10–15). The retinoic acid receptor-related orphan receptor binding elements (RRE) also provide a shared site for RORs (activator) and REV-ERBs (repressor), thereby forming a loop, in which REV-ERBα/β proteins indirectly regulate expression of *Per* genes by suppressing the transcription of *Bmal1* and *E4bp4* through the RRE on their promoters (15–18). In principle, phase entrainment or resetting of the clock must impact the activities or levels of the molecular components of these loops, and the induction of *Per1* gene expression is believed to be a critical step in this process (19–23). Other parallel mechanisms that contribute to entrainment might exist. It would be expected that these unknown input pathways would also impinge on the loops described above.

The phase of the molecular clocks in peripheral tissues are influenced by various external and/or endogenous signals. Numerous endogenous neural, humoral, and metabolic factors, including glucocorticoids, insulin, glucose, retinoic acid, and Ang II have been reported to affect circadian gene expression in cultured cell lines (24–28). External time signals such as feeding and physical activity also affect peripheral tissue clocks (29, 30). It is also reported that several medically prescribed drugs (medical external inputs) drastically change clock gene expression (31–34). Because of the diversity of peripheral tissues and cell-types in the body, the existence of autonomous clock and identification of its potential entrainment signal(s), either external (e.g., light, food intake) or internal (hormonal, neuronal, body temperature), need to be empirically defined on a cell type-by-cell type basis.

In the present study, we focused our attention to evaluating the basic circadian clock properties of adrenal ZG cells. The zona glomerulosa, termed ZG, is a specific component of the adrenal gland cortex. The ZG cells are located in the outermost zone of the cortex and responsible for synthesizing and secreting the steroid hormone aldosterone (35). As a critical element of the renin–angiotensin–aldosterone system (RAAS), these cells express the aldosterone-synthesizing enzyme (AS, or Cyp11b2) under the control of Ang II and play a role in blood pressure homeostasis (36). We previously found that mice lacking the core circadian clock components *Cry1* and *Cry2* exhibit hyperplasia of ZG cells and resultantly show salt-sensitive hypertension phenotype (37). However, it is still not known (remains unexplored) whether ZG cells possess endogenous circadian oscillators, and if so, what their phase (time) entrainment signals are. This current situation contrasts to the extensive research on the circadian aspect of the adrenal gland in the hypothalamic-pituitary-adrenal (HPA) axis (38–41). There are several adrenal-cortex *in situ* hybridization data in the literature, which suggest for the circadian gene expression in the region of ZG layer (38, 42); however, direct evidence to show the rhythmicity of ZG cells is missing. Within this paradigm, in the present study, we have demonstrated the presence of autonomous clock in ZG cells and tested their potential entrainment capacity to Ang II and Ang II receptor antagonist (CV11974) using different cell culture systems. Isolated adrenal explants of *Per2^Luc^* mice, dissociated ZG cells from *Per2-dluc* rats, and a related human adrenocortical cell line H295R, were used in this study.

## 2 Materials and methods

### 2.1 Animals

*Per2*-*dluc* transgenic rats (43), *Per2^Luc^* knock-in mice (44), and *Clock* ^Δ19/Δ19^ mice (45) were bred and genotyped as described previously (46–48). Animals were housed under a regular 12-h light/12-h dark (LD) cycle, maintained at 22 ± 2°C, with free access to food and water. All animal experiments were conducted in compliance with the Ethical Regulations of Kyoto University and performed under protocols approved by the Animal Care and Experimentation Committee of Kyoto University.

### 2.2 Bioluminescence recordings from adrenal slice culture

Adrenals from *Per2^Luc^* mice (male, 8–12 weeks old) were harvested between ZT5–7 and were sliced into a section of 0.3 mm thickness (49) and separately cultured on a Millicell membrane (PICMORG50, Millipore) with serum-free minimum essential medium (MEM) containing 20 mM HEPES, 36 mM glucose, 100 units/ml penicillin, 100 μg/ml streptomycin with 1% ITS+ Premix (BD Biosciences), and 1 mM D-luciferin (Promega), in 35-mm dish. The bioluminescence from the cultured adrenal slice was measured with a highly sensitive cryogenic CCD camera (600 series, Spectral Instruments) coupled to a microscope (Axiovert 135TV, Carl Zeiss) at 35°C. Successive images were acquired every 20 min with an exposure time of 20 min. Background noise was removed from the images by applying a median filter (Metamorph Software, Molecular devices) to the whole stack of pictures, before measuring luminescence intensity from the area of interest. Luminescence intensity data were filtered by a low-pass filter with a cutoff threshold of 72 cycles/day to eliminate stochastic ultradian oscillations. Where indicated, Ang II (Peptide institute) was applied to culture medium at the final concentration of 100 nM. Phase shifts were calculated as the time-interval between the second and third peaks minus the time-interval between the first and second peaks.

### 2.3 Primary culture of rat glomerulosa cells

The rat glomerulosa cells were obtained from adrenal glands of male rats weighing 200–250 g (7–8 weeks old) and isolated according to the method previously established by Payet et al. (50, 51). In brief, *Per2*-*dluc* rats were sacrificed at ZT5–7 and adrenal glands were immediately removed and dissected. The capsules were separated from fasciculata-reticularis by manual compression and incubated for 20 min at 37°C in oxygenized MEM (4 capsules/ml) containing 2 mg/ml collagenase and 125 units/ml deoxyribonuclease. After incubation, cells were disrupted mechanically, filtered through Cell Strainer (100 μm, BD Biosciences), and centrifuged for 10 min at 300*g*. The cell pellet was resuspended in OPTI-MEM medium supplemented with 2% fetal bovine serum, 100 units/ml penicillin, and 100 μg/ml streptomycin (52–54). The cells were then plated in 35-mm dishes (4 capsules/dish) and cultured with repeated medium changes at 24-h intervals for 3 days before experiments.

### 2.4 Transcriptional enhancer reporter assay

H295R cells (ATCC, CRL-2128) were cultured in DMEM/F12 supplemented with 2.5% Nu serum (BD Biosciences) and 1% ITS premix (BD Biosciences) as described previously (47). Cells were transfected with the following reporter plasmids using Lipofectamine LTX (Thermo Fisher Scientific): (i) pGL4.25 *PER1* CRE×3-WT-Luc2CP, in which a tandem repeat of the sequence corresponding to the human *PER1* CRE with flanking sequences (positions –1950 to –1921; 5′-TTC TTC CGC TTT GAC GTC ACT GCT GTC TCC-3′) was cloned into [luc2CP/minP] (Promega), (ii) pGL4.25 *PRE1* CRE×3-Mut-Luc2CP, which is the same as (i) except that CRE sequences were mutated to 5′-TCACATAA-3′, (iii) pGL4.25 *E4BP4* NBRE-like×3-WT-Luc2CP, in which a tandem repeat of the sequence corresponding to the human *E4BP4* NBRE-like (5′-TGACCTTG-3′) with flanking sequences (positions +4452 to +4481) was cloned into pGL4.25, (iv) pGL4.25 *E4BP4* NBRE×3-WT-Luc2CP, in which a tandem repeat of the human *E4BP4* NBRE (5′-TGACCTTT-3′) with flanking sequences (positions +4845 to +4874) was cloned into pGL4.25, and (v) pGL4.25 *E4BP4* NBRE×3-Mut-Luc2CP, which is the same as (iv) except that the human *E4BP4* NBRE sequences were mutated to 5′-TGA ATTCT-3′. Bioluminescence recording was performed a day after transfection.

### 2.5 Adeno-associated virus-mediated circadian clock reporter assay

A luciferase reporter (Luc2P) driven by a mouse or human *Per2* promoter sequence (positions –1670 to +53 for mouse; –1840 to +108 for human) was inserted between the inverted terminal repeat (ITR) sequences in pAAV-MCS2 plasmid (Addgene, Plasmid #46954) to obtain pAAV-*mPer2*-*Luc2P* or pAAV-*hPER2*-*Luc2P*. Recombinant AAV-DJ vectors were produced with a triple-transfection helper-free method as described (55). The day after AAV transduction, cells were split and equally plated into 35-mm dishes containing medium supplemented with 1 mM D-luciferin.

### 2.6 Bioluminescence recordings from cultured cells

Bioluminescence recording was performed using a dish-type luminometer AB-2550 Kronos Dio (ATTO) under 5% CO_2_ atmosphere at 37°C. Luminescence was measured at 20-min intervals, with an exception of 30-min intervals in pharmacological experiments (due to drug application during intervals between measurements). To evaluate dose dependency, CV11974 was administrated at a concentration of 0.001, 0.01, 0.1, and 1 μM. Where indicated, cells were treated with Ang II (100 nM), CV11974 (1 μM) or PD123319 (1 μM, Abcam). For enhancer activity reporter assay, values were normalized to the luminescence intensity that was recorded 30 min before Ang II stimulation. For single cell level recording, successive images were acquired with a highly sensitive cryogenic CCD camera (600 series, Spectral Instruments) with a 20 min exposure time for each picture (3 pictures/h). A median filter was applied to all images to eliminate cosmic-ray-induced background noise. Cell viability test and morphological inspection verify that the cells treated with CV or PD (each at 1 μM) for 24 h remained viable and morphologically unaffected (**Supplementary Figure S1**). Trypan blue extraction assay was used for viability test (56). The half-life of CV and PD in H295R culture is not known (57).

### 2.7 Immunoblotting and quantitative RT-PCR

Cells were lysed in Laemmli buffer and immunoblotting was performed according to our standard method (58) with antibodies against NGFIB (Santa Cruz, sc-5569, 1:500 dilution), E4BP4 (Santa Cruz, sc-9549, 1:1000), and β-actin (Sigma Aldrich, A5441, 1:1000). The data were normalized to the expression levels of β-actin. For qRT-PCR, cells were harvested in TRIzol reagent (Thermo Fisher). Total cell RNA was extracted using RNeasy micro kit (Qiagen) and converted to complementary DNA with SuperScript VILO cDNA synthesis kit (Thermo Fisher). Quantitative real-time PCR was achieved using the Platinum SYBR Green qPCR SuperMix-UDG with ROX (Thermo Fisher) with StepOnePlus system (Applied Biosystems). The data were normalized to a non-oscillatory housekeeping gene, *RPLP0* mRNA levels. The primer sets used in this study included: *PER1*, Fw: 5′-GCA TCT CAG CGG AGC TCA CA-3′, Rv: 5′-GAG GCT GTA GGC AAT GGA ACT G-3′, *PER2*, Fw: 5′-GTG CAG CTC CAC CCT AGT GA-3′, Rv: 5′-GAT TTT CCT GCT CCA TGG GTT GAT G-3′, *CRY1*, Fw: 5′-AGC AAA CTC ACC TGT TGA AGC AAG G-3′, Rv: 5′-GCT GCA ACA GTA TTC CTC CTG AAT G-3′, *BMAL1*, Fw: 5′-AGT CTG TCT TCA AGA TCC TCA ACT AC-3′, Rv: 5′-CTG GAA GTC CAG TTT TTG CAT CTA TG-3′, *DBP*, Fw: 5′-GAA CCC GAC CCA GCT GAT CT-3′, Rv: 5′-CTT GGC TGC CTC GTT GTT CTT GT-3′, *E4BP4*, Fw: 5′-CCC GAG AGC AGG AAC ACG AT-3′, Rv: 5′-ACC CTA TCT ATG TGT GTA GGA GAA C-3′, and *RPLP0*, Fw: 5′-ATG CAG CAG ATC CGC ATG T-3′, Rv: 5′-TTG CGC ATC ATG GTG TTC TT-3′. For temperature entrainment, cells were subjected to 24-h temperature cycles with a 12-h warm phase (37°C) and a 12-h cold phase (33°C) for three days, and then released into constant 37°C conditions.

### 2.8 Immunocytochemistry

Isolated rat glomerulosa cells were seeded on poly-D-lysine-coated coverslips and cultured for 12 h. Cells were fixed with acetone for 10 min at –20°C, immediately rehydrated in 0.1 M phosphate buffered saline (PBS) for 5 min, and incubated with mouse anti-CYP11B2 antibody (Millipore, MAB6021, 1:100) in PBS containing 0.3% Triton X-100 (PBX) for 5 days at 4°C. After washing with PBX, the samples were incubated with Alexa594-conjugated donkey anti-mouse IgG (Thermo Fisher, 1:5000) for 12 h at 4°C. Nuclei were visualized by staining with 4′,6′-diamino-2-phenylindole (DAPI).

### 2.9 *In situ* hybridization

Radioisotopic *in situ* hybridization was performed with following gene-specific probes: for *Agtr1a*, nucleotides 1387–2138 (GenBank, NM_177322); for *Agtr1b*, nucleotides 1298–2015 (NM_175086); for *Cyp11b2*, nucleotides 1744−2456 (S85260). The corresponding cDNA fragment was cloned and used as a template for the generation of riboprobes. The riboprobes were radiolabeled with [^33^P]UTP (PerkinElmer), using a standard protocol for the cRNA synthesis. *In situ* hybridization was performed as described (37). Briefly, paraformaldehyde fixed tissues were frozen and sectioned at a thickness of 30 μm. Then, the free-floating tissue sections were transferred through 4 × SSC, proteinase K (1 μg/ml, 0.1 M Tris-HCl [pH 8.0]; 50 mM EDTA) for 15 min at 37°C, 0.25% acetic anhydride in 0.1 M triethanolamine for 10 min, and 4 × SSC for 10 min. The sections were then incubated in the Denhardt’s hybridization buffer containing 55% formamide, 10% dextran sulfate, 10 mM Tris-HCl (pH 8.0), 1 mM EDTA, 0.6 M NaCl, 0.2% N-laurylsarcosine, 500 μg/ml tRNA, 0.25% SDS, 10 mM dithiothreitol (DTT) and radiolabeled riboprobes for 16 h at 60°C. Following a high-stringency posthybridization wash, the sections were treated with RNase A. Air-dried sections were exposed to X-ray films (Kodak Biomax).

### 2.10 Data and statistical analysis

Cosinor analysis and Rayleigh’s uniformity test were performed using Python 3.8. Western blot band intensities were quantified using ImageJ software. All statistical analyses were performed using GraphPad Prism 8, using the statistical tests for each figure as indicated in the legend.

## 3 Results

### 3.1 Rhythmic PER2 expression in the adrenal ZG region

**Figure 1:**
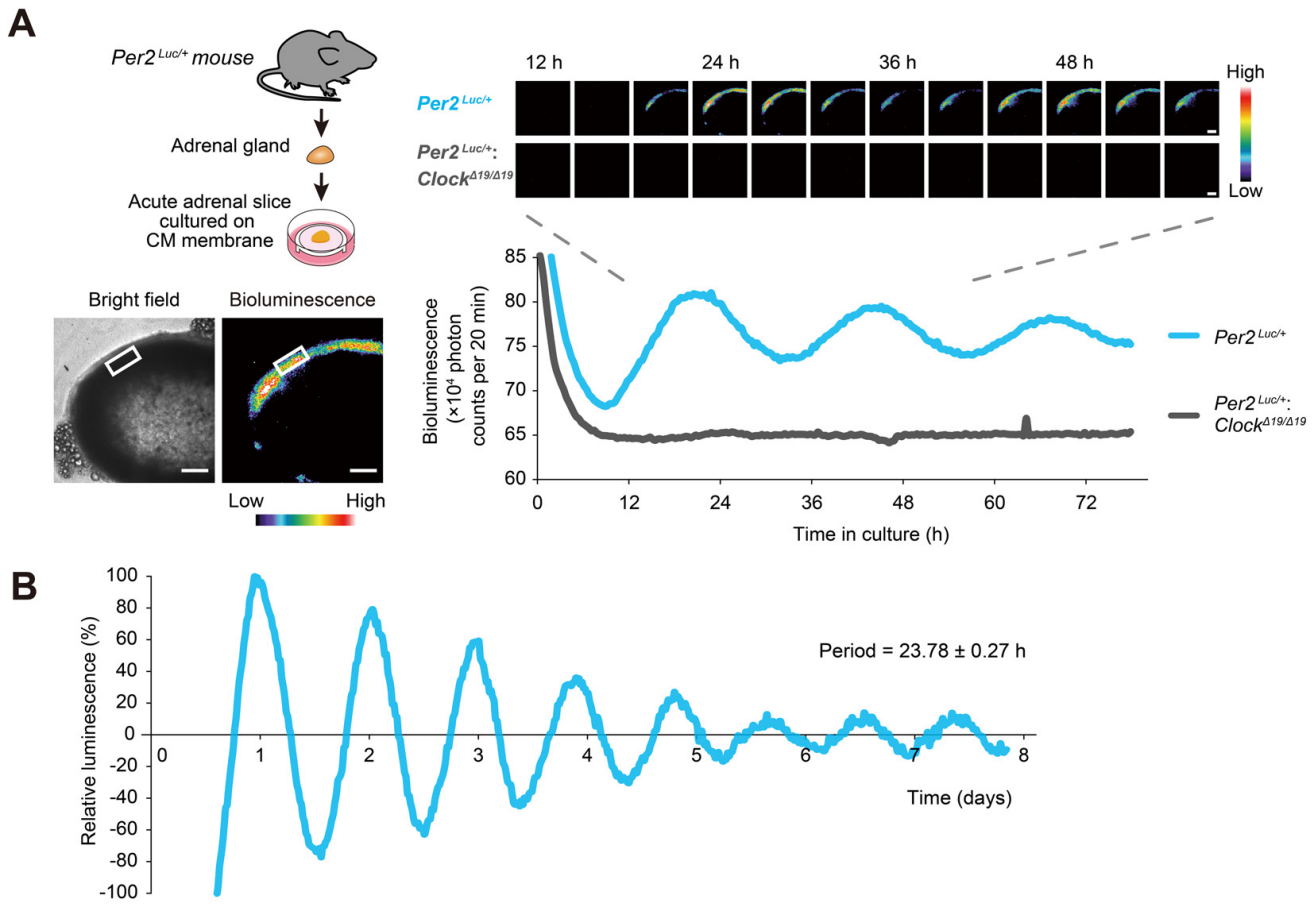
Circadian oscillations exhibited by adrenal ZG cells. **(A)** Time-lapse images of circadian PER2::LUC bioluminescence obtained from *Per2^Luc^* ^/^ *^+^* and *Per2^Luc^* ^/^ *^+^*: *Clock^Δ19^* ^/^ *^Δ19^* mouse adrenal slices. Intensity was traced from a region of the adrenal cortex outer layer containing ZG cells (white boxes) over 80 h. Bioluminescence intensity is represented in pseudo-color scale. Scale bars, 200 μm. **(B)** Representative long-term bioluminescence recording of the ZG in *Per2^Luc^* ^/^ *^+^* adrenal from five independent experiments. Data were detrended by 24-h moving average. The maximum bioluminescence was set to 100%.

To test expression of the core clock gene in mouse adrenal, we used isolated adrenal explants from *Per2^Luc^* knock-in mice, which facilitate the analysis of circadian expression of Per2 in tissues and cells (44). Bioluminescence imaging revealed abundant PER2::LUC luminescence located mostly in the outer layer of the adrenal cortex, where ZG cells are located (**Figure 1A**); These observations are similar to those reviewed by other researchers (59). We found that ZG cells in *Per2^Luc^* explants exhibit persistent luminescence oscillations for more than a week in culture, with a period length of approximately 24 hours (23.78 ± 0.27 h, mean ± SEM, *n* = 5 biologically independent slices) (**Figure 1B**). The PER2::LUC luminescence rhythms were abolished in adrenals from *Per2^Luc^*; *Clock^Δ19/Δ19^* mice (**Figure 1A**), indicating that these rhythms are generated by the endogenous circadian clock mechanism.

### 3.2 Circadian oscillations exhibited by dispersed cell culture of ZG cells

**Figure 2:**
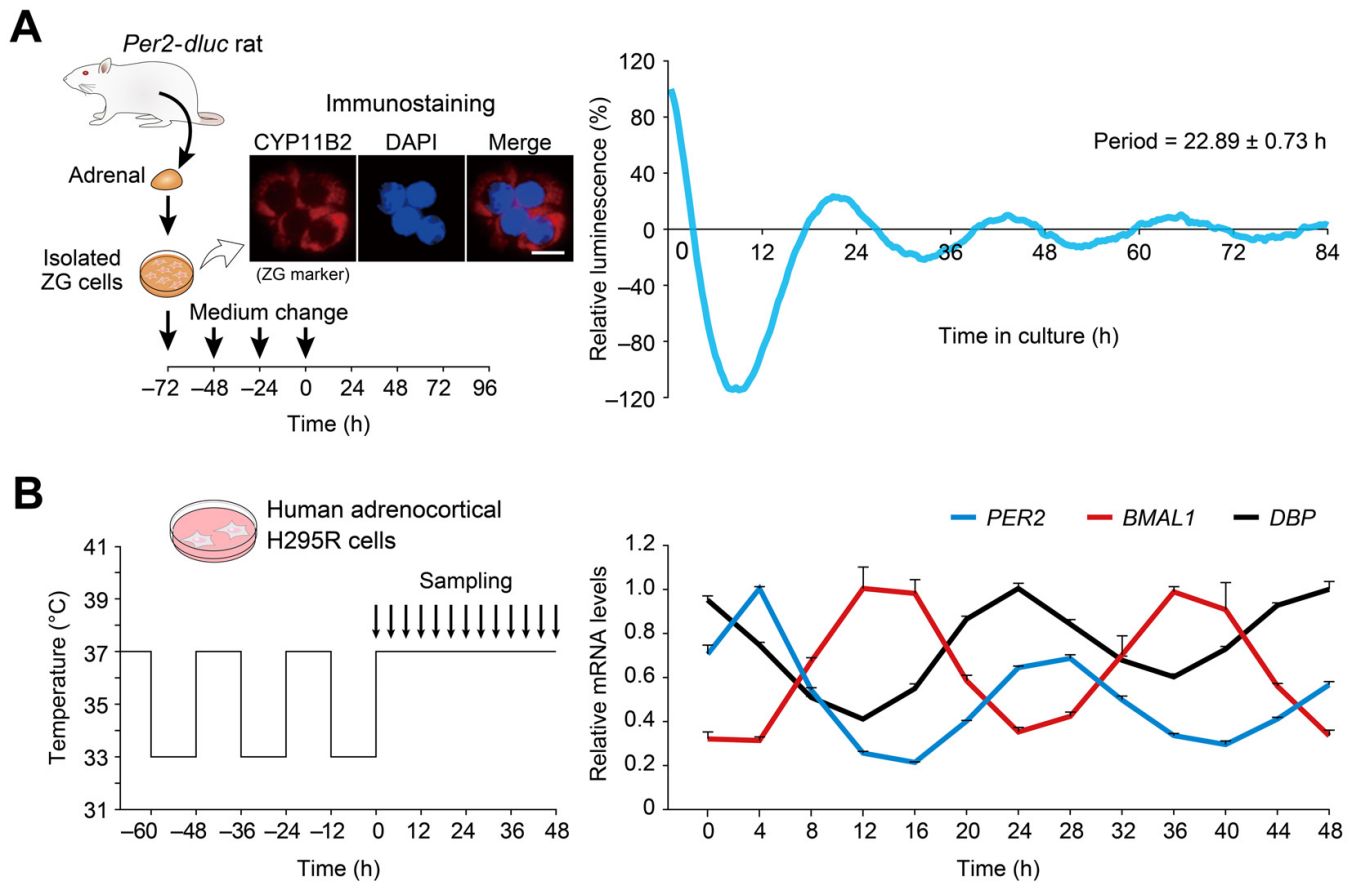
Circadian oscillations displayed by dispersed cell culture of ZG cells and human H295R adrenocortical cells. **(A)** Representative *Per2*-*dluc* bioluminescence recording of dissociated rat primary ZG cells. ZG cells were entrained by 24-h interval medium changes and then released into constant conditions with no medium change. The data were detrended by 24-h moving average and plotted from the last medium change. Immunocytochemistry for CYP11B2 confirmed the isolation of ZG cells from rat adrenal glands. Scale bar, 10 μm. Periods were determined from three measurements. **(B)** Circadian oscillation of clock genes in human adrenocortical H295R cells. Cells pre-synchronized to 37°C/33°C temperature cycles were harvested under constant 37°C temperature conditions. The data were normalized to *RPLP0*. The peak mRNA values of each gene were set to 1. *n* = 3 biological replicates per timepoint. Values are means ± SEM.

Next, we tested dissociated cell culture of ZG cells (**Figure 2A**). For this particular purpose, we used *Per2*-*dluc* rat (43), since isolation of primary ZG cells has been established in rats (50, 51). Immunocytochemistry confirmed that approximately 90 percent of the cells isolated were immunolabelled for the ZG cell-marker Cyp11b2, an enzyme responsible for aldosterone production, verifying enrichment of ZG cells as reported (54) (**Figure 2A**). The freshly isolated rat ZG cells were maintained with repeated medium changes at 24-h intervals for 3 consecutive days (for establishment of primary cell culture) and subsequently maintained under constant culture conditions without medium change for an additional 96 h for luminescence tracing. Under these conditions, sustained circadian bioluminescence rhythms were observed (**Figure 2A**), indicating that cultured ZG cells can act as a circadian oscillator.

As an extension of the above investigation, we also asked whether the human adrenocortical cell line H295R exhibits circadian oscillation (**Figure 2B**). H295R cells have been widely utilized as a model system for studying ZG (60), including aldosterone biosynthesis (61, 62). However, it is currently not known whether this human model cell line shares similar clock gene expression.

Temperature is known to entrain (or synchronize) cellular clocks of many tissues *in vitro* (63–65). We cultured H295R cells for 3 days with a temperature cycle (12 h at 37°C, 12 h at 33°C) and placed them in constant temperature conditions (37°C). Cells were harvested every 4 h over a 48-h period to study circadian variations. Quantitative RT-PCR analysis (**Figure 2B**) revealed that the expression levels of *PER2* and *BMAL1*—representatives of two major feedback loops within the clockwork—showed high-amplitude circadian oscillations. *PER2* exhibited its circadian peak expression 8 h earlier than *BMAL1*, and *DBP* mRNA expression fluctuated in the opposite phase of *BMAL1*—a phasic relationship consistent with the current molecular model of the circadian clock (7–9). Our data indicate that mouse and rat ZG cells and human H295R cells all retain a functional clockwork.

### 3.3 Ang II can induce phase shifts of the adrenal ZG clock

**Figure 3:**
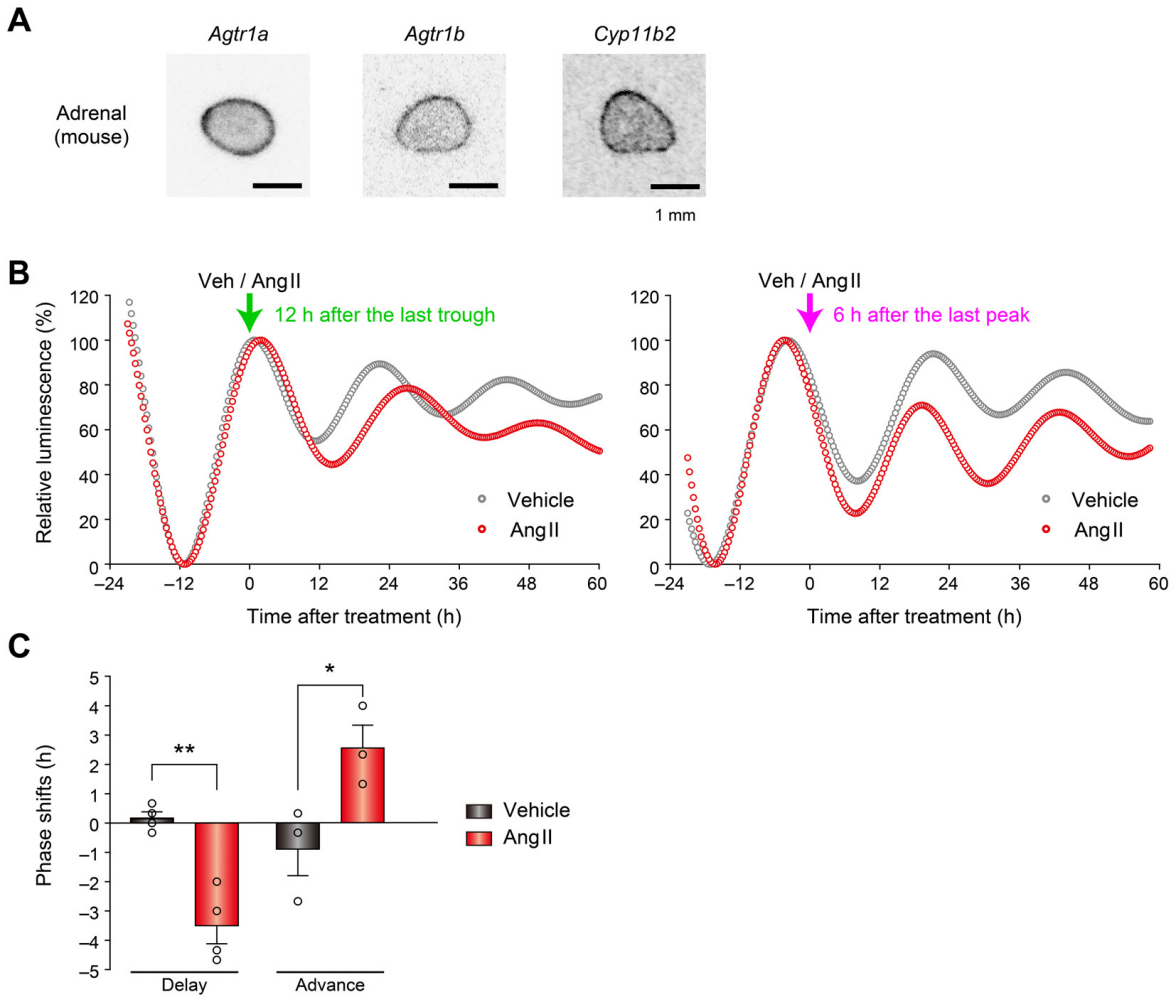
Ang II elicits phase-dependent phase shifts of the adrenal ZG clock. **(A)** Autoradio-graphs showing expression of *Agtr1a*, *Agtr1b*, and *Cyp11b2* in the mouse adrenal section. **(B)** Phase shifts of PER2::LUC rhythm after Ang II treatment in adrenal slices. Arrows indicate the time of Ang II or vehicle administration. Luminescence of ZG was traced. The peak and trough values were adjusted to 100 and 0, respectively. **(C)** Quantification of the magnitude of phase shifts shown in **(B)**. Phase delays and advances are plotted as negative and positive values, respectively. *n* = 3–4 slices per condition. Values are means ± SEM. *P < 0.05, **P < 0.01, unpaired two-sided Student’s t test.

Because Ang II is a potent physiological regulator of ZG function (35), we hypothesized that Ang II may modulate circadian rhythms in ZG. *In situ* hybridization using radioisotope-labeled probes for the Ang II type 1 receptor (AT1R) subtypes *Agtr1a* and *Agtr1b* confirmed expression of AT1Rs in the mouse ZG (**Figure 3A**), further prompting us to test Ang II’s effect on ZG clock. When Ang II was administered to cultured adrenal slices at different phases of luminescence, it caused phase-dependent phase-shifts (delay versus advance) (**Figure 3B**): administration at the luminescence peak (12 h after the trough) significantly delayed the phase, whereas administration 6 h after the peak advanced the phase significantly (*P* = 0.001 for phase delay, *P* = 0.045 for phase advance, unpaired two-sided Student’s t test, **Figure 3C**). These results demonstrate that mouse ZG contains an Ang II-regulatable circadian clock.

### 3.4 Ang II has the ability to reset circadian oscillation of H295R cells

**Figure 4:**
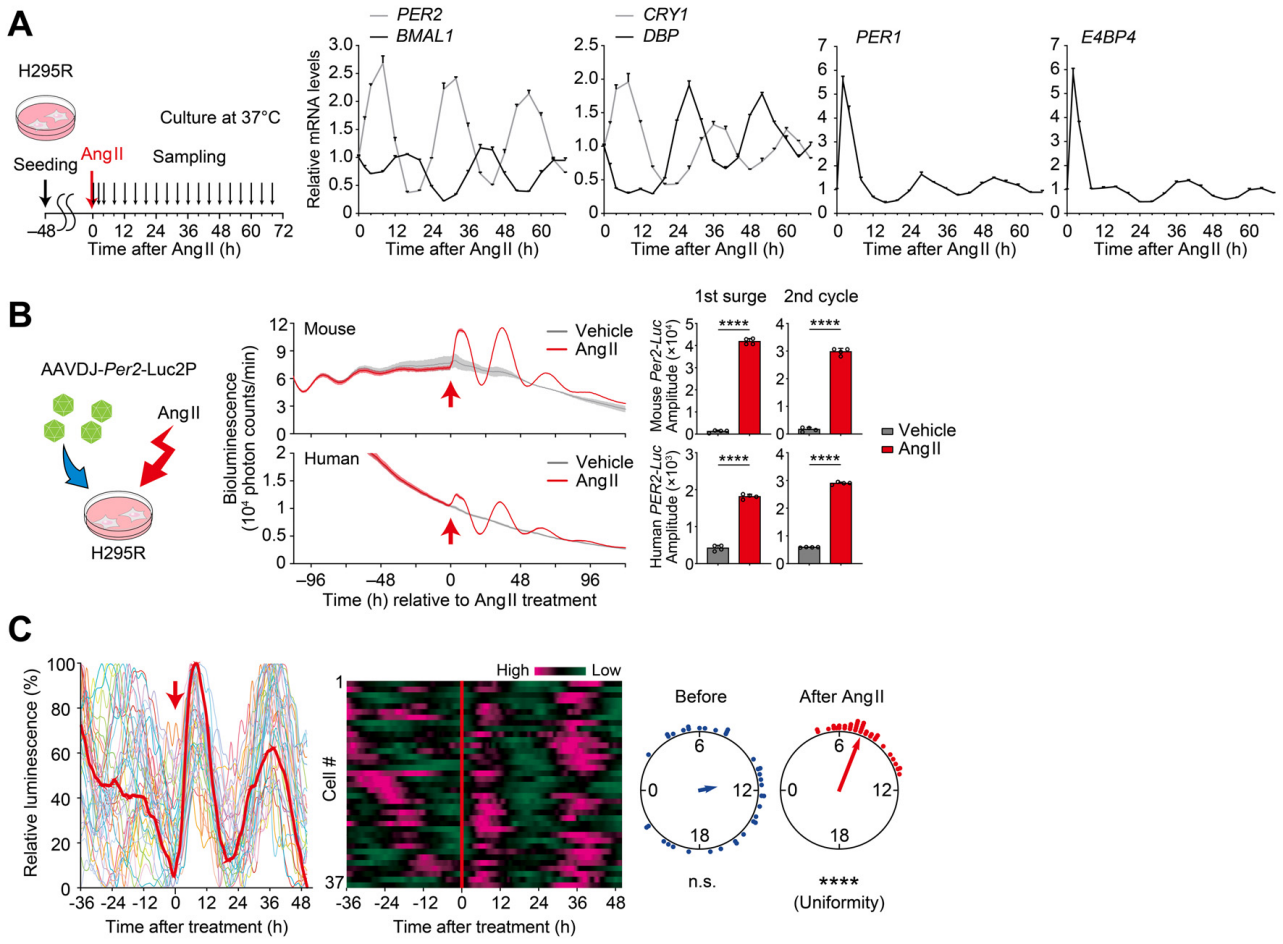
Ang II resets circadian rhythms in H295R cells. **(A)** Circadian expression profiles of representative core clock genes and clock-controlled genes in H295R cells. Cells were treated with Ang II at Time 0 and were harvested at 0, 2, and every 4 hour over a 68-h period. Values are means ± SEM from *n* = 3 biological replicates per timepoint. Results of cosinor analysis of the clock gene expression profiles are available in **Supplementary Table S1**. **(B)** A cartoon for viral infection to H295R cells and luminescence traces of cells harboring a luciferase reporter under the control of human or mouse *Per2* promoter. Arrows indicate the time of Ang II or vehicle administration. Bar graphs illustrate the amplitude of the first surge and second cycle of luminescence following Ang II administration. Data are the means ± SD from *n* = 4 biologically independent experiments. **(C)** Single-cell bioluminescence tracing in H295R cells expressing mouse *Per2-Luc* reporter. Heat maps show individual cellular luminescence, where magenta corresponds to peak bioluminescence and green to trough. Rayleigh plot shows phase distribution of acrophase of individual cells before and after Ang II treatment. *n* = 37 cells. Statistics in **(B)**, unpaired two-sided Student’s t test; in **(C)**, Rayleigh’s uniformity test. ****P < 0.0001.

The human adrenocortical H295R cells were also treated with Ang II to test for the resetting effect of Ang II stimulation (**Figure 4**). To do this, H295R cells were seeded onto 24-well plates and cultured for 2 days to confluency; then, after Ang II treatment, cells were harvested at 0, 2, and every 4 hour over a 68-h period (*n* = 3 replicates; total 57 wells, **Figure 4A**). Quantitative RT-PCR analysis revealed rhythmical expression of *PER1*, *PER2*, *BMAL1*, *CRY1*, *DBP*, and *E4BP4* that continued over 3 circadian cycles after Ang II treatment (cosinor analysis, available in **Supplementary Table S1**). There was an acute and transient upregulation of *PER1* mRNA expression immediately after Ang II stimulation. In addition, a similarly prominent (~ 6 fold) upregulation of mRNA expression was also identified for *E4BP4*, while no appreciable elevation was observed following vehicle administration (see **Supplementary Figure S2**). These observations, taken together, indicate that Ang II stimulation has the ability to reset or (re)initiate circadian clock gene expression in H295R cells.

In order to facilitate real-time tracing of circadian clock gene expression, we next employed a luciferase reporter system. We generated AAV encoding a luciferase under the *PER2* promoter from either human or mouse origin and transduced it to H295R cells. Then, luminescence was traced both before and after Ang II stimulation, in culture (**Figure 4B**). Luminescence rhythmicity gradually dampened before Ang II treatment. Following Ang II administration, a significant and high-amplitude surge was observed, along with sustained luminescence oscillations in subsequent cycles (*P* < 0.0001, for the first surge and second circadian cycle, vs vehicle control, **Figure 4B**). Single-cell-level analysis of circadian rhythms was further performed by CCD video recording of H295R cells before and after Ang II treatment (**Figure 4C**). We observed that upon Ang II treatment, bioluminescence rhythms of individual cells were immediately resynchronized. This was evident as the vertical alignment of peak bioluminescence across all traced cells in the heatmap of cellular rhythms after Ang II treatment. These data illustrate the phase-resetting of individual cells [see also ref. (66)].

### 3.5 The AT1R blocker mitigates Ang II-induced clock resetting in H295R cells

**Figure 5:**
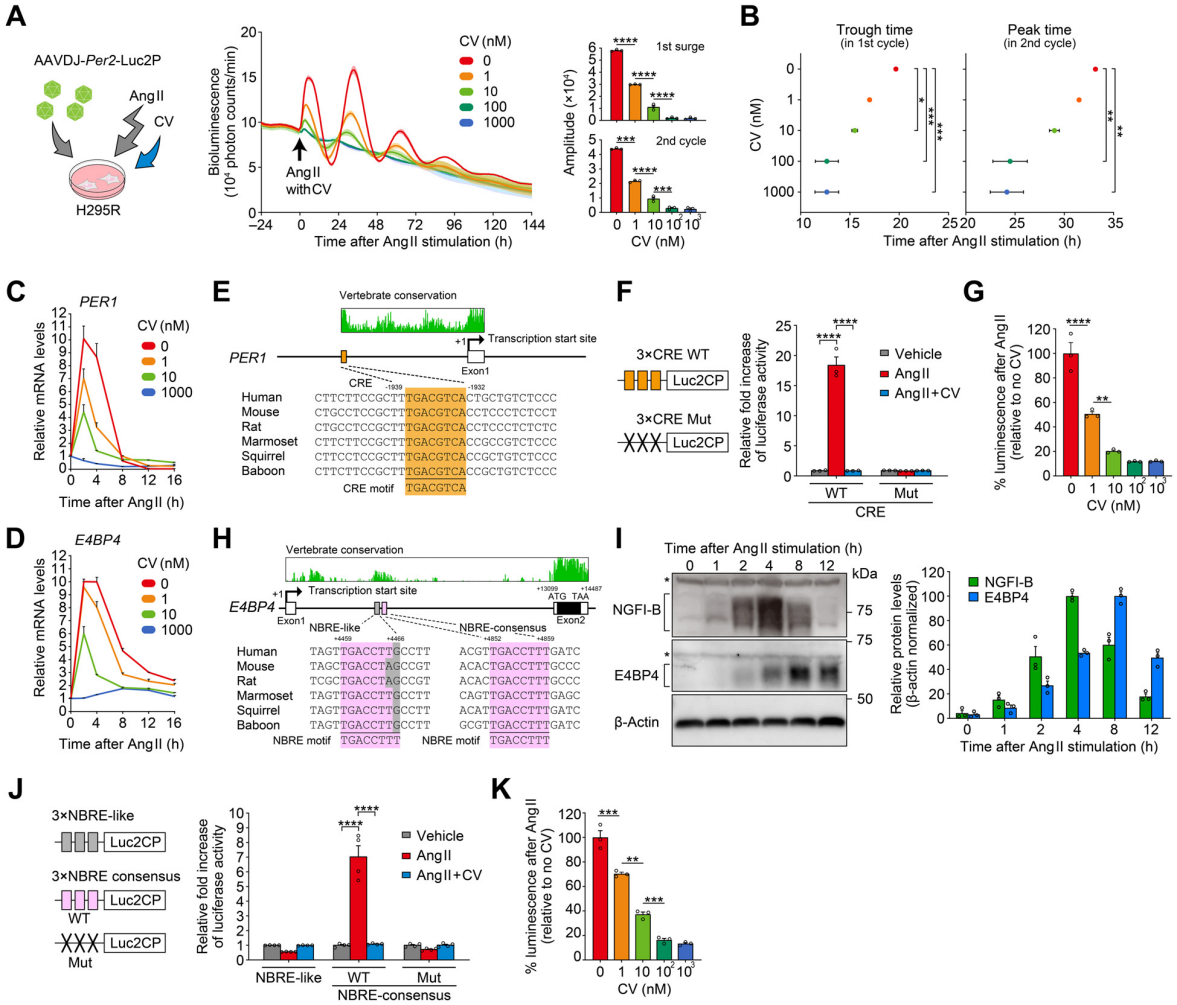
Ang II-induced clock resetting through a mechanism involving upregulation of *PER1* and *E4BP4*. **(A, B)** Effects of increasing concentrations of CV on Ang II-induced circadian luminescence in H295R cells. Cells were transduced with a luciferase reporter under the control of mouse *Per2* promoter. Arrows indicate the time of Ang II/CV treatment. Bar graphs in **(A)** illustrate the amplitude of the first surge and second cycle of luminescence following Ang II administration with different doses of CV. Plots in **(B)** show the first trough and second peak phase of the luminescence rhythm following Ang II treatment. *n* = 3 biological replicates for each CV concentration. Traces in **(A)** are expressed as means ± SD. **(C, D)** Effects of CV on Ang II-induced *PER1* and *E4BP4* mRNA expression in H295R cells. *n* = 3 biological replicates per datapoint. **(E)** Sequence alignment of CRE located in the promoter of *PER1*. The sequences of CRE are compared among mammalian species along with the consensus CRE motif (5′-TGACGTCA-3′). Genomic positions, relative to the transcription start site (+1), are indicated along with the conservation scores obtained from the UCSC Genome Browser (https://genome.ucsc.edu/). **(F)** Relative reporter activities of CRE×3-Luc2CP and its mutant (Mut, TCACATAA). Cells received vehicle, Ang II, or Ang II plus CV (1 µM). *n* = 3 biological replicates. **(G)** Dose-dependent effects of CV on CRE reporter activity after Ang II stimulation. *n* = 3 biological replicates for each CV concentration. **(H)** Sequence alignment of two candidate NRBEs, both located in the intron 1 of *E4BP4*. The sequences of NBRE-like (*left*) and NBRE-consensus (*right*) are aligned among species with the consensus NGFI-B binding motif (5′-TGACCTTT-3′ or 5′-AAAGGTCA-3′). *E4BP4* consists of two exons. **(I)** Immunoblots showing the protein expression profiles of NGFI-B and E4BP4 after Ang II stimulation. Bar graph shows protein quantification data (*n* = 3 biological replicates). β-Actin serves as a loading control. Asterisks indicate nonspecific bands. Uncropped blots are available in **Supplementary Figure S5**. **(J)** Relative reporter activities of NBRE-like×3-Luc2CP, NBRE-consensus×3-Luc2CP, and its mutant (Mut, TGAATTCT). *n* = 4 biological replicates. **(K)** Dose-dependent effect of CV on NBRE-consensus reporter activity after Ang II stimulation. *n* = 3 biological replicates for each CV concentration. Statistics in **(A, B, G, K)** were one-way ANOVA followed by Tukey’s multiple comparisons test; in **(F)** and **(J)**, two-way ANOVA followed by Sidak’s multiple comparison test. Values are means ± SEM; *P < 0.05, **P < 0.01, ***P< 0.001, ****P < 0.0001.

CV11974 (CV) is a receptor antagonist specific to AT1R. We found that CV treatment dose-dependently suppressed the rhythm resetting of H295R cells (**Figure 5A**). In this experiment, a *Per2-Luc* reporter was also virally introduced into H295R cells. As the CV dose increased (0, 1, 10, 100, and 1000 nM), the *Per2-Luc* expression induced by Ang II decreased accordingly. This resulted in a similar dose-dependent attenuation of the *Per2*-*Luc* rhythm amplitude in the following circadian cycles (**Figure 5A**). Moreover, CV shortened the duration of the initial surge of the *Per2-Luc* expression, which in turn influenced the phase of the *Per2-Luc* rhythms in the subsequent circadian cycles (**Figure 5B**; compare phases across different CV doses). We observed that the Ang II type 2 receptor inhibitor PD did not affect either the amplitude or phase of the *Per2*-*Luc* rhythm in the same H295R cells (**Supplementary Figure S3**). Without Ang II, CV treatment had no effect on the *Per2-Luc* expression (**Supplementary Figure S4**), consistent with a conjecture that AT1R is not basally activated in the culture conditions that we used for H295R cells (67–69).

Furthermore, we observed that the magnitude of the initial induction of endogenous *PER1* and *E4BP4* mRNA expression in Ang II-treated H295R cells was also decreased by CV treatment in a dose-dependent manner (**Figures 5C, D**). Considering that CV is one of the most commonly used clinical drugs for hypertension treatment, its dose-dependent effects on clock gene expression imply a previously unstudied potential action of the drug [for known actions of CV, see (70)].

### 3.6 Potential *cis*-elements for Ang II-induced *PER1* and *E4BP4* transcriptional upregulation

It has been reported that *PER1* is acutely responsive to various stimuli through the cAMP-response element (CRE) located in its promoter and that Ang II causes upregulation of CRE- mediated gene transcription in H295R cells (71–76). Consistently, the isolated human *PER1* CRE sequence (5′-TGACGTCA-3′) significantly increased reporter transcription upon Ang II stimulation, and CV treatment dose-dependently reduced this activation (see **Figures 5E−G** for *PER1*). However, CRE has not previously been implicated in the regulation of *E4BP4* (77).

Previous studies investigating Ang II-downstream signaling in H295R cells (61, 78–80) revealed two independent, DNA-binding transcription factor families — ATF/CREB family and NGFI-B family — that mediate transient transcription of a different set of genes upon Ang II stimulation. Because CRE sequence (ATF/CREB-binding motif) is not present in the human *E4BP4* locus, we rather propose that the NGFI-B-responsive element (NBRE, 5′-TGA CCTTT-3′) may play a role in the Ang II/CV-dependent mRNA induction of *E4BP4* (**Figures 5H−K**). *E4BP4* consists of two exons. Sequence conservation analysis revealed that the intron 1 contains a highly conserved segment (+4283 to +5181, relative to the transcription start site) harboring two putative NBRE sequences at +4459 to +4466 (NBRE-like) and +4852 to +4859 (NBRE-consensus), but no CRE sites (see **Figure 5H**). Western blots (**Figure 5I**) confirmed acute accumulation of NGFI-B, noticeable as early as 2 h after Ang II treatment, consistent with previous reports (61, 68). E4BP4 protein levels peaked around 8 h after Ang II treatment (**Figure 5I**). Subsequently, we assessed the potential *cis*-element function using reporter vectors containing tandem repeats of the putative *cis*-element with flanking regions (NBRE-consensus×3-luc and NBRE-like×3-luc) (**Figure 5J**). In H295R cells, Ang II significantly increased the reporter activity of NBRE-consensus, and CV effectively blocked this effect. Contrastingly, neither Ang II nor CV had any effect on the NBRE-like sequence, indicating the sequence specificity of Ang II-induced transactivation in H295R cells. We confirmed that mutation of the NBRE-consensus sequence abolished its Ang II-dependent enhancer activity (**Figure 5J**, Mut). Finally, we applied CV at the same various doses as used in **Figure 5A**. We found that the upregulation of NBRE-consensus reporter activity was dose-dependently reduced by CV, in a manner similar to the changes observed in endogenous *E4BP4* mRNA expression in H295R cells (**Figures 5D, K**). We thus propose that the NBRE sequence in intron 1 may be involved, at least partly, in the Ang II-regulated transcription of *E4BP4* in H295R cells.

## 4 Discussion

There still remain several cell types whose circadian clock functionality has yet to be tested. Here, we showed that the circadian clock resides in cells in adrenal ZG and its related human H295R cell line. Our data indicate that they are genetically-encoded, self-sustained and cell-autonomous circadian clock, and responsive to external Ang II stimulation. Consistent with the reaction to Ang II, ZG cells were found to express Ang II receptor type I subtypes, *Agtr1a* and *Agtr1b*. Furthermore, we observed that the type I receptor specific blocker CV modulates the level of clock gene expression in Ang II-stimulated H295R cells. Given that CV is widely used for hypertension treatment, its dose-dependent effect on clock gene expression may warrant consideration as a potential additional action of this drug (discussed below).

In the present study, we provide evidence that Ang II-regulatable cell-autonomous circadian oscillators reside in the adrenal gland ZG cells. Time-lapse bioluminescence microscopy of isolated adrenal glands from *Per2^Luc^* reporter mice revealed abundant and robustly cycling Per2::Luc luminescence in the adrenal ZG cells, which was completely abolished in *Clock* mutant mice (**Figure 1A**). These data thus demonstrate that the oscillations in ZG cells are driven by the endogenous, genetically encoded circadian clock. Self-sustainable oscillations are also presented by dissociated rat ZG cells and human H295R cells (**Figure 2**). Furthermore, we found that circadian rhythms in ZG can be modulated by external Ang II treatment in culture; Ang II treatment produced phase-dependent phase-shifts (**Figure 3**). Using H295R cells, we also showed phase resetting that acutely occurs in response to Ang II stimulation. Our single-cell data suggest that the immediate phase-resetting of individual cellular rhythms results in the emergence of overt rhythms at the cell population level [cf. ref. (66)] (**Figure 4**). Altogether, our results provided evidence to show the presence of Ang II-responsive, cell-autonomous circadian clocks in adrenal ZG and its related H295R cells.

The Ang II type 1 receptors have been localized in vascular smooth muscles, adrenal gland, kidney, heart, brain, platelets, and placenta (81). Previous *in vitro* studies with cultured cells indicate that Ang II signaling through AT1R has the ability to phase reset the clock gene expression in cardiomyocytes and vascular smooth muscle cells (28, 82). However, with regard to *in vivo* relevance, no previous studies demonstrate the effect of endogenous Ang II on circadian clock function in peripheral tissues. Particularly, it is still unclear whether Ang II functions as an endogenous entrainment signal for peripheral tissues such as the ZG cells we identified in this study. In this regard, it is noteworthy that a modest diurnal rhythm of plasma Ang II concentration was reported for humans and rats (83–87) while plasma angiotensinogen levels do not exhibit significant circadian oscillation (88). Thus, *in vivo* role of Ang II as a systemic entrainer remains to be studied in our future research. Clinically, Ang II itself is not used for therapeutic purposes. By contrast, CV is one of the most widely prescribed drugs for clinical treatment of hypertension and related cardiovascular diseases such as heart failure, myocardial infarction and diabetic nephropathy (89–93). Thus, exogenously given (or taken) CV may rather warrant attention from a practice point of view. It is not known or considered if CV affects the ZG circadian clock, in addition to its authorized actions, in clinical use. However, given our observations of its dose-dependent effect on clock gene expression, CV introduction with once-daily dosing (at a fixed timepoint or a more temporally random scheme) may have an additional influence on the clock phase in ZG; CV may enhance the rhythmicity of clock gene expression at certain regimes, while at other intervals, it could potentially diminish that rhythmicity. Although purely hypothetical, these implications may be of use in considering the repertoire of actions associated with this drug.

*E4bp4* (also known as *Nfil3*) has been recognized as a key regulator in the field of immune system and neurobiology, as well as in the circadian clock biology. In CD4^+^ T cells, *E4bp4* is upregulated by chronic antigen stimulation and mediates upregulation of production of IL-10 and IL-13, which helps prevent excessive immune responses (94). In natural killer (NK) cells, *E4bp4* activation enhances the cytotoxic activity, contributing to cancer suppression (95, 96). In epilepsy, reduced expression of *E4bp4* in neurons causes an increased incidence of seizure (97). *E4bp4* activation in microglia restrains microglial cell activation via ERK1/2 signaling and alleviates delirium-associated cognitive decline (98). In terms of circadian biology, on the other hand, the direct functional role of *E4bp4* is still not fully understood (99–101); knockout studies show that *E4bp4* is dispensable for maintaining circadian rhythms of mouse locomotor activity (99, 101), while knockdown of *E4bp4* lengthened the circadian period in cultured rat-1 cells (100), indicating that *E4bp4* is not an essential requirement of the central clock function. Nevertheless, it is still possible that *E4bp4* may contribute to peripheral clock time-keeping and/or time-resetting system of certain tissue/cell types. Yoshitane et al. demonstrated that in *in vitro* cultured mouse embryonic fibroblast cells, acidic stress (pH 7.0 → pH 6.6) caused acute upregulation of *E4bp4* mRNA-protein expression and showed that genetic deficiency of *E4bp4* resulted in abrogated phase-resetting due to acid stress in the cells (101), indicating the potential of *E4bp4* as a phase-modulator for peripheral cells. In our study, we observed that *E4BP4* mRNA-protein expression is strongly and acutely upregulated by Ang II to the levels almost comparable to those of acute induction of *PER1* in H295R cells (**Figure 4A**). *PER1* induction has long been considered to be involved in phase resetting of cells (19–23); however, remaining phase-resetting capacities of *PER1* knockout cells (U2OS cells) to several resetting stimuli in culture (serum shock and forskolin) (102) and the fact that grafting *Per1* knockout embryonic fibroblasts into wild-type mice results in synchronization with the host’s rhythm *in vivo* (103) support the idea that *Per1* induction is not the sole control point for resetting the clock and rather suggest that the responsible mechanism relies on a broader network of changes beyond *Per1* induction (101, 104). In this light, exploration of the extent to which *E4BP4* contributes to Ang II-induced clock resetting will be a subject of our future study (12, 105); additional investigations using *PER1* and *E4BP4* double deficient cells and KO mice will be required to assess the mechanism of Ang II-induced molecular clock resetting.

Collectively, in the present study, we have elucidated the presence of Ang II-responsive molecular clock in rodent adrenal ZG cells and adrenocortical H295R cells. Because of the diversity of peripheral tissues and cell-types, the existence of autonomous clock and identification of its potential entrainment signal(s), either external (e.g., light, food intake) or internal (hormonal, neuronal, body temperature), need to be empirically defined on a cell type-by-cell type basis. Our studies showing the presence of autonomous clock in ZG cells and its potential entrainment capacity to Ang II and its receptor antagonist CV (a potential exogenous stimulus) may provide a basis to understand *in vitro* circadian properties of ZG cells as a possible component of various peripheral clocks.

There are a number of limitations in our study. Firstly, we did not test all entrainment methods for each culture condition. Temperature entrainment was tested for H295R cells because it is a common method for cell culture entrainment (26, 65, 106–108) and regarded as a physiologically relevant entrainment cue in the body (109, 110). In the rat primary ZG cell culture, however, temperature cycles (changes) negatively affect cell viability upon establishment of primary culture; it requires repeated medium exchange during the initial development (at 37°C). We leveraged these conditions, instead of applying temperature cycles, for entrainment. In our study, we focused on the effect of Ang II and did not test for the effects of other hormones such as mineralocorticoid and glucocorticoid on the ZG circadian clock. Given the proximity between ZG and the zona fasciculata (ZF), where glucocorticoids are produced, a question remains as to the interaction between ZG clock and ZF clock. Secondly, only male rodents were used in the present study, while H295R cells are derived from a female patient with adrenocortical carcinoma (111). Considering known sex-dependent differences in adrenal functions, including stress responsivity (112), tissue renewal (113) and AT1R expression (114–116), further *in vivo* studies will be required to investigate sex-derived differences in clock function in the ZG, including the rhythmicity of AT1R availability. Thirdly and lastly, Ang II-responsive element(s) responsible for *E4BP4* induction remains unclear. We found a functional NBRE site in the intron 1 of *E4BP4*. However, the extent to which this element contributes to Ang II-responsiveness of *E4BP4 in vivo* (in H295R cells) needs to be verified by cells harboring specific mutation on this site. We do not exclude the possibility that other *cis*-regulatory elements that we could not identify in this study may also contribute to the up-regulation of *E4BP4* in response to Ang II as well as to other stimuli.

## Data Availability

The raw data supporting the conclusions of this article will be made available by the authors, without undue reservation.

## Supplementary material

Supplementary Table 1 Characteristics of circadian mRNA expression rhythms in H295R cells, related to **Figure 4A**. MESOR (midline-estimating statistic of rhythm), a rhythm-adjusted 24-hour mean; amplitude, half of total predictable change in rhythm, defined by rhythmic function fitted to data; acrophase, peak time of a fitted cosine curve; period, the time span of a complete rhythmic cycle estimated from the fitted cosine function; R-factor, goodness-of-fit parameter for curve fits.
