# Supplementary material for: Identification of angiotensin II-responsive circadian clock gene expression in adrenal zona glomerulosa cells and human adrenocortical H295R cells
Source: Front Endocrinol (Lausanne). 2025 Mar 26;16:1525844. doi: 10.3389/fendo.2025.1525844 (PMC11978646; doi:10.3389/fendo.2025.1525844)
Supplement: Supplementary Table 1 — Characteristics of circadian mRNA expression rhythms in H295R cells, related to Figure 4A . MESOR (midline-estimating statistic of rhythm), a rhythm-adjusted 24-hour mean; amplitude, half of total predictable change in rhythm, defined by rhythmic function fitted to data; acrophase, peak time of a fitted cosine curve; period, the time span of a complete rhythmic cycle estimated from the fitted cosine function; R-factor, goodness-of-fit parameter for curve fits. [file DataSheet1.pdf]

Supplementary Figure 1

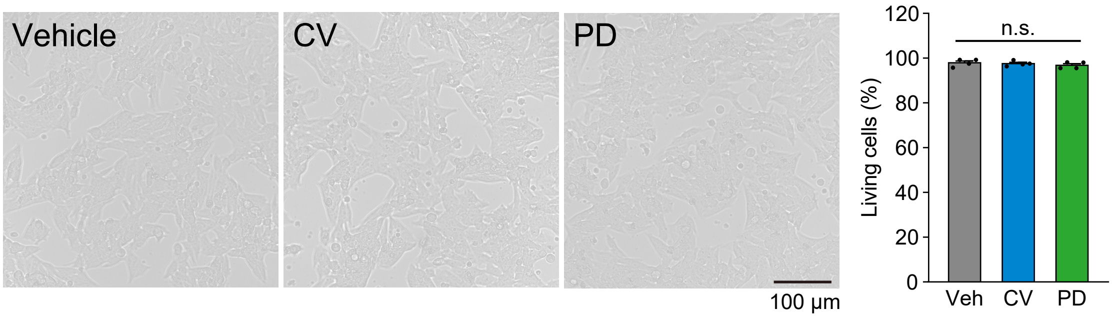

Fig S1 | Viability of H295R cells treated with AT1R blockers. Representative cell images and cell viability of H295R cells treated with or without CV (1 µM) or PD (1 µM) for 24 h. Cell viabllity was assessed by trypan blue dye exclusion assay. *n* = 4 biological replicates. Data are the means ± SEM. Statistical significance was assessed using one-way ANOVA followed by Tukey's multiple comparisons test. n.s., not significant.

Supplementary Figure 2

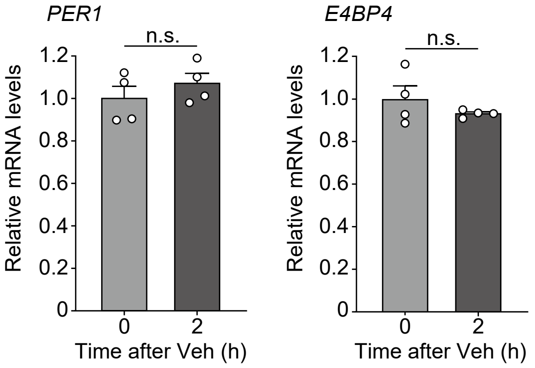

Fig S2 | Absent effect of vehicle treatment on *PER1* and *E4BP4* mRNA expression in H295R cells. H295R cells were treated with vehicle, and after 2 h, total RNA was isolated from the cells and the levels of mRNA for *PER1* and *E4BP4* were determined by using qRT-PCR. All the values (means ± SEM, $n = 4$) were normalized to the levels of *RPLP0*, and values for each gene at time zero were set equal to 1. Statistical significance was assessed using unpaired two-sided Student's t test. n.s., not significant.

Supplementary Figure 3

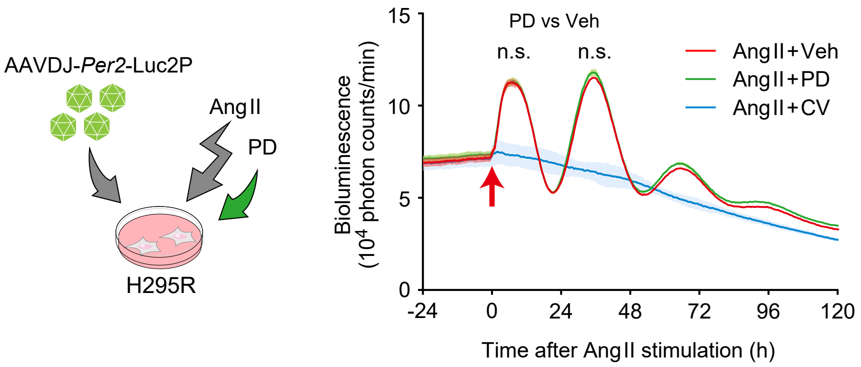

Fig S3 | Absent effect of Ang II type 2 receptor blocker PD on Ang II-induced circadian bioluminescence in H295R cells. Experiments were performed as described in Fig 4B using virally infected H295R cells. Traces indicate luminescence from cells harboring a luciferase reporter under the *Per2* promoter. PD, CV or vehicle (Veh) was administered together with Ang II at the time point indicated by the red arrow. The data for the treatment with Ang II and Veh (Ang II + Veh) are identical to those plotted in Fig 4B. Data are the means ± SD. Statistical significance was assessed using one-way ANOVA followed by Tukey's multiple comparisons test. n.s., not significant, PD vs. Veh for the first and second peak expresison of luminescence.

Supplementary Figure 4

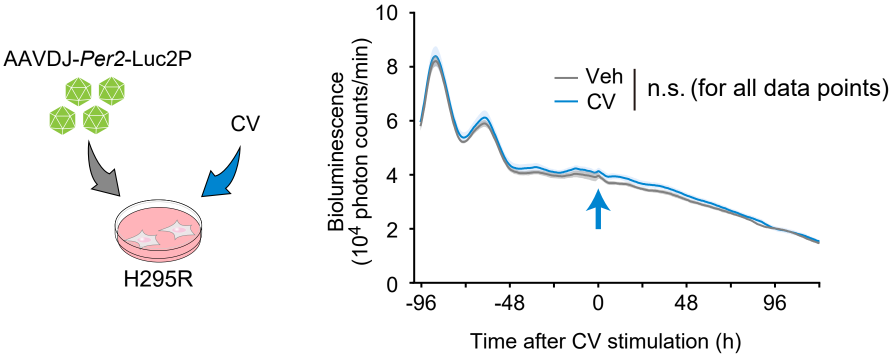

Fig S4 | Absent effect of CV on *Per2-Luc* expression in H295R cells without Ang II coadministration. Experiments were performed as described in Fig 4B using virally infected H295R cells. Traces indicate luminescence from cells harboring a luciferase reporter under the *Per2* promoter. CV or vehicle (Veh) was administered at the time point indicated by the blue arrow. Data are the means ± SD. *n* = 4 biologically independent traces. Statistical significance was assessed using two-way ANOVA followed by Sidak's multiple comparison test. n.s., not significant.

Supplementary Figure 5

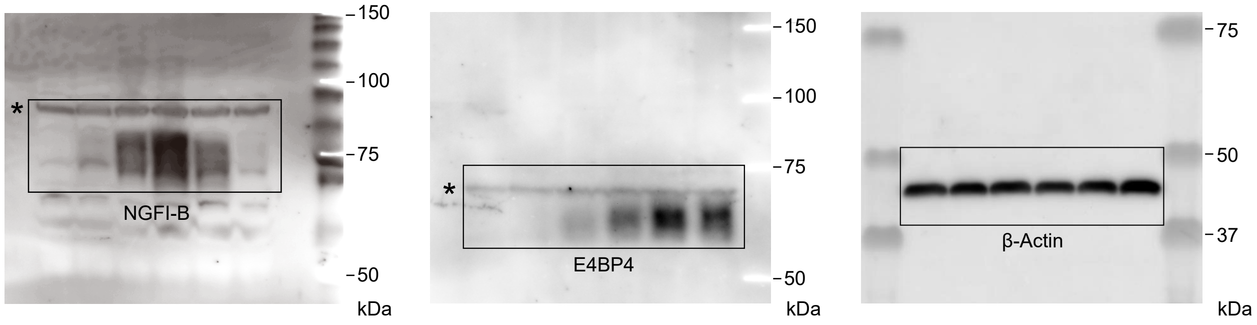

Fig S5 | Full version of western blots shown in Fig 5I. Asterisks indicate nonspecific bands.

Supplementary Table 1

Table S1 | Characteristics of circadian mRNA expression rhythms in H295R cells, related to Fig 4A. MESOR (midline-estimating statistic of rhythm), a rhythm-adjusted 24-hour mean; amplitude, half of total predictable change in rhythm, definied by rhythmic function fitted to data; acrophase, peak time of a fitted cosine curve; period, the time span of a complete rhythmic cycle estimated from the fitted cosine function; R-factor, goodness-of-fit parameter for curve fits.

	MESOR	Amplitude	Acrophase	Period (h)	R-factor
PER2	1.40	0.88	7.47	24.42	0.98
BMAL1	0.69	0.41	18.56	25.06	0.97
CRY1	0.93	0.36	12.79	23.99	0.96
DBP	1.21	0.52	3.93	24.02	0.95
PER1	1.14	0.35	5.33	23.52	0.92
E4BP4	0.87	0.36	15.80	24.78	0.90
